# DNA methylation-based patterns for early diagnostic prediction and prognostic evaluation in colorectal cancer patients with high tumor mutation burden

**DOI:** 10.3389/fonc.2022.1030335

**Published:** 2023-01-13

**Authors:** Hao Huang, Weifan Cao, Zhiping Long, Lei Kuang, Xi Li, Yifei Feng, Yuying Wu, Yang Zhao, Yinggang Chen, Peng Sun, Panxin Peng, Jinli Zhang, Lijun Yuan, Tianze Li, Huifang Hu, Gairui Li, Longkun Yang, Xing Zhang, Fulan Hu, Xizhuo Sun, Dongsheng Hu

**Affiliations:** ^1^ Department of General Practice, The Affiliated Luohu Hospital of Shenzhen University Health Science Center, Shenzhen, Guangdong, China; ^2^ Department of Epidemiology, Public Health School of Harbin Medical University, Harbin, China; ^3^ Department of Epidemiology and Health Statistics, School of Public Health, Shenzhen University Health Science Center, Shenzhen, China; ^4^ Department of Gastrointestinal Surgery, Shenzhen Hospital, National Cancer Center/Cancer Hospital, Chinese Academy of Medical Sciences and Peking Union Medical College, Shenzhen, China; ^5^ Department of Chronic Disease Control and Prevention, Shenzhen Nanshan Center for Chronic Disease Control, Shenzhen, China; ^6^ Department of Epidemiology and Health Statistics, Fujian Provincial Key Laboratory of Environment Factors and Cancer, School of Public Health, Fujian Medical University, Fuzhou, China

**Keywords:** TMB, DNA methylation, colorectal cancer, immunotherapy, TCGA

## Abstract

**Background:**

Immune checkpoint inhibitor (ICI) therapy has proven to be a promising treatment for colorectal cancer (CRC). We aim to investigate the relationship between DNA methylation and tumor mutation burden (TMB) by integrating genomic and epigenetic profiles to precisely identify clinical benefit populations and to evaluate the effect of ICI therapy.

**Methods:**

A total of 536 CRC tissues from the Cancer Genome Atlas (TCGA) with mutation data were collected and subjected to calculate TMB. 80 CRC patients with high TMB and paired normal tissues were selected as training sets and developed the diagnostic and prognostic methylation models, respectively. In the validation set, the diagnostic model was validated in our in-house 47 CRC tissues and 122 CRC tissues from the Gene Expression Omnibus (GEO) datasets, respectively. And a total of 38 CRC tissues with high TMB from the COLONOMICS dataset verified the prognostic model.

**Results:**

A positive correlation between differential methylation positions and TMB level was observed in TCGA CRC cohort (r=0.45). The diagnostic score that consisted of methylation levels of four genes (*ADHFE1*, *DOK6*, *GPR75*, and *MAP3K14-AS1*) showed high diagnostic performance in the discovery (AUC=1.000) and two independent validation (AUC=0.946, AUC=0.857) datasets. Additionally, these four genes showed significant positive correlations with NK cells. The prognostic score containing three genes (*POU3F3*, *SYN2*, and *TMEM178A*) had significantly poorer survival in the high-risk TMB samples than those in the low-risk TMB samples (*P*=0.016). CRC patients with low-risk scores combined with TMB levels represent a favorable survival.

**Conclusions:**

By integrating analyses of methylation and mutation data, it is suggested that DNA methylation patterns combined with TMB serve as a novel potential biomarker for early screening in more high-TMB populations and for evaluating the prognostic effect of CRC patients with ICI therapy.

## Introduction

Colorectal cancer (CRC) is one of the most common primary malignant tumors and the second leading cause of cancer-related mortality worldwide (1). Although recent advances in diagnostic and therapeutic methods for managing CRC have greatly improved the survival of early CRC patients (CRCs), the majority of them are diagnosed in the middle and advanced stages of the disease (2). The 5-year survival rate was shown to be only 14% in late-stage CRCs, but over 90% in early-stage CRCs (3). If a diagnosis can be made at any stage before the advanced stage, patients will suffer from less tumor burden and endure less treatment; hence, early diagnosis and suitable treatments are crucial to improve the survival rate of CRC patients.

In addition to traditional therapies, immune checkpoint inhibitor (ICI) treatments have proven promising treatments for CRCs, including anti-PD-1, anti-PD-L1, and anti-CTLA4 (4). These anti-tumor immunotherapies could normalize patients’ own immune systems in the tumor microenvironment, possibly transforming cancer into a chronic disease (5, 6). PD-L1 expression, microsatellite instability (MSI), and deficient mismatch repair (dMMR) have emerged as major predictive markers for the efficacy of ICI therapy (7); however, not all the CRC patients predicted by PD-L1 expression and MSI benefit from ICI therapy. Moreover, only approximately 15% of CRCs are MSI with high PD-L1 expression (8, 9). Alongside PD-L1 expression and MSI, tumor mutation burden (TMB), due to its relatively high positive screening rate (10, 11), was also considered a promising effective biomarker in distinguishing CRCs potentially responsive to ICI therapies. In MSI CRCs, high TMB could favor the infiltration of immune cells, demonstrating stronger anti-tumor immune response and better efficacy of ICI therapy (12). Even in microsatellite stable (MSS) CRCs with DNA polymerase epsilon mutations, high TMB could also favor heavy infiltration of immune cells and represent better immune responses and efficacy of therapy (13, 14). Nevertheless, the clinical application of TMB is limited due to the high cost of whole exome sequencing (WES) and the indefinite thresholds for high TMB (15, 16). Finding an effective biomarker in combination with TMB may therefore be a better way to precisely identify more populations that benefit from CRC ICI therapies.

CRC is a multi-step and complex disease that involves a series of genetic and epigenetic alterations. In terms of molecular subtypes of CRC, CRCs can be classified into three distinct molecular signatures based on integrated DNA methylation and mutation profiling, including CpG island methylator phenotype (CIMP) 1, characterized by MSI (80%) and BRAF mutations (53%) and rare KRAS and TP53 mutations (17, 18). CRCs can also be further categorized into four consensus molecular subtypes (CMS). Among them, CMS1 represents a hypermutated phenotype, frequently characterized by BRAF mutations, highly enriched in CIMP and MSI (76%) (19–21). DNA methylation may therefore be correlated with tumor mutation in CRC. In addition, aberrant DNA methylation is involved in the initiation and progression of CRCs by reprogramming the epigenetic landscape (22). Recent studies have demonstrated that DNA methylation was an effective predictive marker for clinical benefits of ICI treatment in tumors, including CRC (23, 24). Low-dose decitabine has the function of demethylation, which can increase the expression of immune-related antigens in tumor cells, thereby making tumor cells more easily recognized by the immune system and creating more suitable immunotherapy conditions for CRC patients (24, 25). Moreover, Cai et al. found that there was a significant positive correlation between DNA methylation changes and TMB, which could identify lung cancer patients who have a clinical response to immunotherapy (26). However, the direct correlation between DNA methylation and TMB in CRCs and its impact on immunotherapy has not been explored to date.

In this study, we integrated DNA methylation profiling and tumor mutation data to investigate the direct correlation of DNA methylation with TMB and to discover and better understand novel early diagnostic and prognostic biomarkers for ICI therapies in CRC.

## Materials and methods

### Patients and datasets

A total of 24 CRC patients with high TMB from the Third Affiliated Hospital of Harbin Medical University were included in this study in 2017. The study was ethically approved by the Institutional Ethics Committee of Harbin Medical University and written informed consent was collected from each study subject. After obtaining informed consent, CRC tissues were obtained for WES, and CRC (N=24) and normal tissues (N=23) were performed for DNA methylation profiling. Moreover, all publicly available databases used in this study were obtained from The Cancer Genome Atlas (TCGA) (https://portal.gdc.cancer.gov/repository) and the Gene Expression Omnibus (GEO) (https://www.ncbi.nlm.nih.gov/geo) databases. The profiles of somatic mutation data for CRC (N=536) were directly downloaded from the TCGA GDC Data Portal. DNA methylation data of Illumina Human Methylation 450 arrays and the corresponding clinical information of TCGA CRCs were acquired from the UCSC Xena Browser (https://xena.ucsc.edu/) (N=448), including 403 CRC tissues and corresponding 45 adjacent non-tumor tissue samples. In the GEO database, four methylation datasets of the Illumina 450K and 850K EPIC arrays were obtained as a validation set by a systematic search (N = 122): GSE107352 (27), GSE128067 (28), GSE148766 (29), and GSE68060 (30), including 92 CRC tissue samples and corresponding 30 adjacent tissue samples, and a batch correction was conducted using the ComBat method. A methylation dataset of the Illumina 850k EPIC array with three cell-free DNA (cfDNA) blood samples from CRC with metastases liver and four cfDNA samples from healthy controls were acquired from GSE122126 (31). An additional Illumina 850k EPIC array methylation-based dataset (GSE175699) with 44 melanoma patients was obtained to predict the response to ICI therapy (32). Additionally, a total of 38 CRC patients with high-TMB from the COLONOMICS database (https://www.colonomics.org/) containing tumor mutation data, DNA methylation data, and prognosis information to evaluate the prognosis of CRCs. The detailed information from the datasets used in this study is summarized in **Figure S1** and **Table 1**.

**Table 1:** Overview of the datasets used in this study.

Dataset	Source	Assay	Sample type	Number of samples
Discovery set (N=448)				
	TCGA CRC	Infinium 450K	Tissue	Normal=45; CRC=403
Test set				
Test set A (N=122)	GSE107352	Infinium 450K	Tissue	Normal=18; CRC=30
	GSE128067	Infinium 450K	Tissue	Normal=6; CRC=17
	GSE148766	Infinium EPIC	Tissue	CRC=36
	GSE68060	Infinium 450K	Tissue	Normal=6; CRC=9
Test set B (N=47)	Inhouse study	Targeted sequencing	Tissue	Normal=23; CRC=24
Test set C (N=7)	GSE122126	Infinium EPIC	cfDNA	Normal=4; CRC=3
Test set D (N=44)	GSE175699	Infinium EPIC	Tissue	melanoma=44

### WES and DNA methylation data analysis

DNA libraries preparation, exome capture and sequencing, and data processing were conducted by Sz-acegen Biotechnologies (Shenzhen, China). Genomic DNA was captured and amplified with Agilent SureSelect Human All Exon version 6 (Agilent Technologies, USA). Sequencing reads were depicted to the human genome (hg19) by the Burrows‐Wheeler Alignment tool (BWA). According to Genome Analysis Toolkit (GATK), the mapped reads with known deletions were locally compared in order to improve the overall quality of alignment. In TCGA, somatic mutation annotation was processed by VarScan2 for the “Masked Somatic Mutation” data. The somatic mutations in the Mutation Annotation Format (MAF) were conducted by the “maftools” R package for visualization and summarization of MAF files. The TMB was defined in two ways: 1) the number of mutations of each sample in proportion to the size of 33.4 Mb (UCSC Refseq annotations)(Number of mutation/33.4); 2) the total amount of non-synonymous coding somatic mutation (NOMs) per tumor sample containing single nucleotide variations (SNVs) and short insertion or deletion polymorphisms (INDELs) (26). Consistent with the estimating approach of mutational burden from MSK-IMPACT (33), high-TMB and low-TMB groups were defined as the top 20% of TMB CRCs and the bottom 20% of TMB CRCs, respectively. High-TMB and low-TMB CRCs were selected in the following analysis.

Genomic DNA of tumor and adjacent tissues were obtained using the QIAamp DNA Blood Mini kit (Qiagen, Hilden, Germany) and stored at –80°C, and then DNA was modified with bisulfite through the EpiTect Fast DNA Bisulfite Kit (Qiagen, Hilden, Germany) and stored at –20°C based on the manufacturer’s instructions. According to the next-generation sequencing for the analysis of multiple targeted CpG methylation, methylation levels of candidate genes were estimated by Methyl Targeted sequencing (Sz-acegen Biotechnologies Inc., China). The targeted DNA sequences were firstly amplified by the method of Multiplex PCR and the designed DNA fragments were sequenced by Illumina Hiseq 2000. The samples with bisulfite conversion rate < 98% and with high missing rates (> 20%) were filtered out. After the quality control procedures, CRC patients remained for further study. In addition, TCGA CRC methylation data were analyzed by the minfi R package. The methylation level of the CpG sites (CpGs) was expressed as β value and calculated according to the average of all the methylation sites of CpG island located at the promoter region, including translation start sites (TSS)1500, TSS200, and 1st Exon region and 5’ untranslated region (5’ UTR). TCGA CRC methylation data were analyzed by the minfi R package. The differential methylated positions (DMPs) of each sample were identified by the β value of tumor and adjacent normal tissues with a false discovery rate (FDR) q-value < 0.05. Then, the differential methylation regions (DMRs) were identified as the following criteria: 1) the promoter region with more than five CpG sites; 2) differentially methylated between tumor and adjacent tissues, with a mean β value difference of at least 0.2 (Δβ ≥ 0.2). Based on the distribution of DMR, differential methylated genes (DMGs) were subsequently identified. DMGs were defined as a class of genes whose promoter regions overlapped with DMR. A total of 468 DMGs were identified in the promoter regions.

### Construction and validation of methylation-based diagnostic score

According to the DMR results obtained, two algorithms were performed to screen novel diagnostic biomarkers for high-TMB CRC, including the least absolute shrinkage and selection operator (LASSO) logistic regression and support vector machine recursive feature elimination (SVM-RFE) (34, 35). Finally, a four-gene methylation diagnostic risk-score (DRS) was constructed by the logistic regression model in the TCGA discovery set. Four GEO methylation datasets were used to validate and evaluate the robustness of the four-gene methylation DRS in the validation set. In addition, we also verified the diagnostic performance of four-gene methylation DRS in our CRC samples. The GSE122126 methylation dataset was further used to initially evaluate the applicability and generalizability of the four-gene score in cfDNA samples. The receiver operating characteristic (ROC) curve was performed to assess the diagnostic performance of the four-gene model for distinguishing high TMB CRC and adjacent normal tissues or blood samples.

### Construction and validation of methylation-based prognostic score for high-TMB CRC

Additionally, univariate Cox regression analysis was utilized to screen prognostic genes associated with the overall survival (OS) of high TMB CRCs based on the results of 468 DMGs differential methylated genes (DMGs). Then, the high-TMB CRC-related methylation prognostic model was constructed according to the methylation of survival-related genes and their relevant coefficients from multivariate Cox regression analysis. The median value of the prognostic risk score (PRS) was defined as the cut-off value and used to divide high-TMB CRCs into high-risk and low-risk groups. The time-dependent ROC and Kaplan-Meier curves were used to evaluate the prognostic capacity of PRS. Further, univariate and multivariate Cox regression analyses were performed to investigate whether the PRS was an independent predictor of CRCs. The gene set enrichment analysis (GSEA) method based on the KEGG and GO gene sets were used to explore differential methylation genes and related functions in high- and low-risk groups.

### Comprehensive analysis of methylation and immune characteristics and ICI therapy

To identify different immune characteristics of high TMB CRC samples, the methylation data of these samples were analyzed using the “Epigenetic Dissection of Intra Sample Heterogeneity (EpiDISH)” R package to estimate the relative proportions of 6 types of immune cells, including B cells, CD4+ T cells, CD8+ T cells, NK cells, monocytes, and granulocytes (36, 37). We separately assessed the proportions of six tumor-infiltrating cells between two groups in diagnostic and prognostic models and further analyzed the prognostic value of candidate biomarkers in ICI therapy.

### Statistical analysis

All statistical analyses were conducted using the R software version 4.1.2. Spearman correlation coefficient was calculated to evaluate the correlation between DNA methylation and TMB. Principal component analysis (PCA) were used to show the difference and separation between tumor and normal samples in high- and low-TMB. *P* < 0.05 was considered statistically significant.

## Results

### DNA methylation difference between high TMB and low TMB CRCs

**Figure 1:**
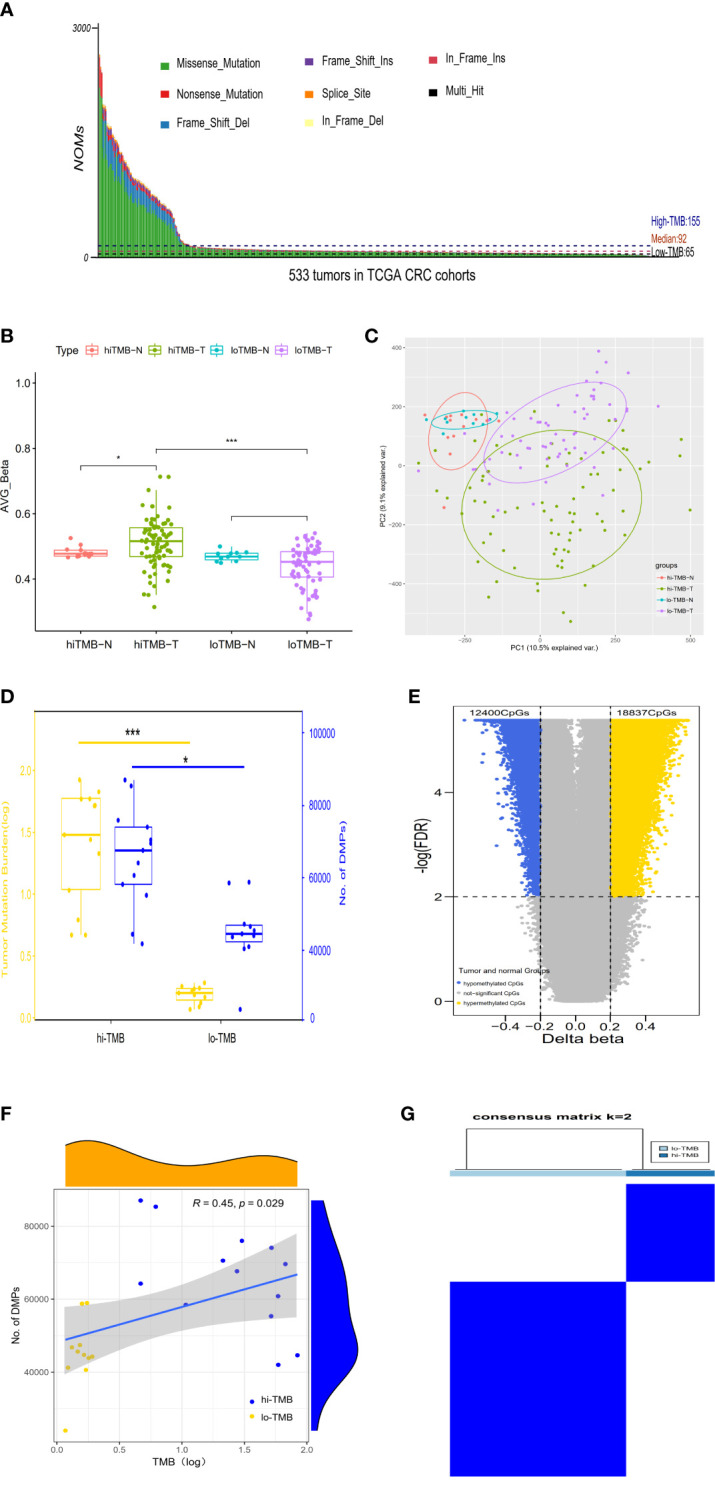
The correlation between differential DNA methylation and TMB in colorectal cancer. **(A)** The number of non-synonymous coding somatic mutations (NOMs) for every CRC patient (represented by the x-axis). Blue/Black lines represent the high/low TMB cutoff value. The samples of above 3000 NOMs were not shown in the figure. **(B)** Bar plot. **(C)** PCA analysis for the average value of all 450K methylation CpG sites in high/low TMB. **(D)** The comparison of differences in DNA methylation between high TMB and low TMB groups; with yellow representing the value of log (TMB) and blue indicating the positions of differential methylation. **(E)** The Volcano plot between delta-beta value (high TMB tumor versus normal) and corresponding –log (FDR) for all 450K methylation CpG sites, with the blue indicating hypermethylated CpGs and the yellow representing hypomethylated CpGs. **(F)** The correlation analysis of differential methylation sites and TMB of CRC patients. **(G)** Consensus clustering of the DNA methylation distinguishing high and low TMB CRC groups of DNA methylation, the rows and columns represent DMPs and samples in the consistent clustering matrix heatmap, respectively. (*P < 0.05; ***P < 0.001).

According to the screening criteria for the high confident somatic mutation (No. of mutation>=5, TMB>=10%) (38), we included 533 CRC samples with mutation data for the following analysis in TCGA dataset, which contains 398 colon and 135 rectum tumor samples. The median number of NOMs per tumor was 92 (ranging from 25 to 10717) when calculating TMB per tumor (**Figure 1A**). According to the approach of mutational burden from MSK-IMPACT, we classified CRCs into high-TMB group (top 20% by TMB, N=107) (155-10717 mutations or 4.6-320.9 mutations/Mb) and low-TMB group (bottom 20% by TMB, N=107) (25-65 mutations or 0.7-1.9 mutations/Mb). To further explore the correlation between DNA methylation and TMB, we selected 80 high-TMB (155–8208 mutations or 4.6–245.7 mutations/Mb) and 65 low-TMB (25–65 mutations or 0.7–1.9 mutations/Mb) tumor samples with both DNA methylation and mutation data, and corresponding 13 high-TMB (156–2803 mutations or 4.7–83.9 mutations/Mb) and 11 low-TMB (39–64 mutations or 1.2–1.9 mutations/Mb) paired adjacent normal samples.

In TCGA CRC dataset, DNA methylation levels in tumor and adjacent normal tissues were measured by the Illumina Infinium HumanMethylation 450k BeadChip platform which cover the methylation status of 485,577 CpGs of the human genome. According to the methylation level of 485,577 sites, the differential global methylation level was higher in tumor (median beta-value of 0.516) than in normal tissues (median beta-value of 0.477) in the high-TMB CRCs, but it was slightly lower in tumor (median beta-value of 0.452) than in normal tissues (median beta-value of 0.468) in the low-TMB CRCs (**Figure 1B**). We further evaluated the global methylation status in tumor and normal tissues by principal component analysis (PCA) of the CpGs, revealing that the distribution of global methylation in tumor tissues differed from the corresponding normal tissues in both high- and low-TMB CRCs (**Figure 1C**); however, compared with low-TMB CRCs, high-TMB CRCs indicated a wider distribution in global methylation patterns. In addition, we conducted differential methylation analyses between high-TMB tumor and matched normal of a single patient, and found that the differential methylation sites were significantly more in high-TMB CRCs (DMPs: 42,004~87,046, median = 67,651) than in low-TMB CRCs (DMPs: 23,971~58,938, median = 44,733) (**Figure 1D**). DMRs were also significantly more in high-TMB CRCs (735 DMRs) than in low-TMB CRCs (265 DMRs) (**Supplementary Table S1**).

In light of so many DMPs in the high-TMB CRCs, we conducted differential methylation analysis between 80 high-TMB CRC tumor and 13 adjacent normal tissues and calculated the Δβ value (**Figure 1E**). From over 450,000 informative probes, 31,237 methylation variable positions (MVPs) were identified according to the criteria of |Δβ| > 0.2 and FDR *q*-value < 0.01, indicating less than 6% of the total CpGs. The top 3,000 MVPs are shown in **Supplementary Table S2**. We identified 18,837 hypermethylated CpGs and 12,400 hypomethylated CpGs in the high-TMB CRCs. DMPs comparing the tumor and matched adjacent normal tissues for each CRC patient were used to estimate their relationship with TMB by Spearman correlation. The DMPs of CRC were significantly associated with TMB (Spearman correlation coefficient = 0.45, *P-*value =0.029) (**Figure 1F**). To further identify whether DMPs can distinguish high-TMB CRCs from low-TMB CRCs, we clustered 640 most significant DMPs from 24 CRCs with paired tumor and normal tissues by the K-Means consensus method with the following parameters: s.d. > 0.2 between high- and low-TMB CRCs, s.d. < 0.2 in high- or low-TMB CRCs, |Δβ| > 0.2, and FDR *q*-value < 0.05 (**Supplementary Table S3**). The consensus clustering of 640 DMPs could significantly distinguish high- and low-TMB CRCs (**Figure 1G**).

### Construction and validation of the methylation-based diagnostic model in high TMB CRCs

**Figure 2:**
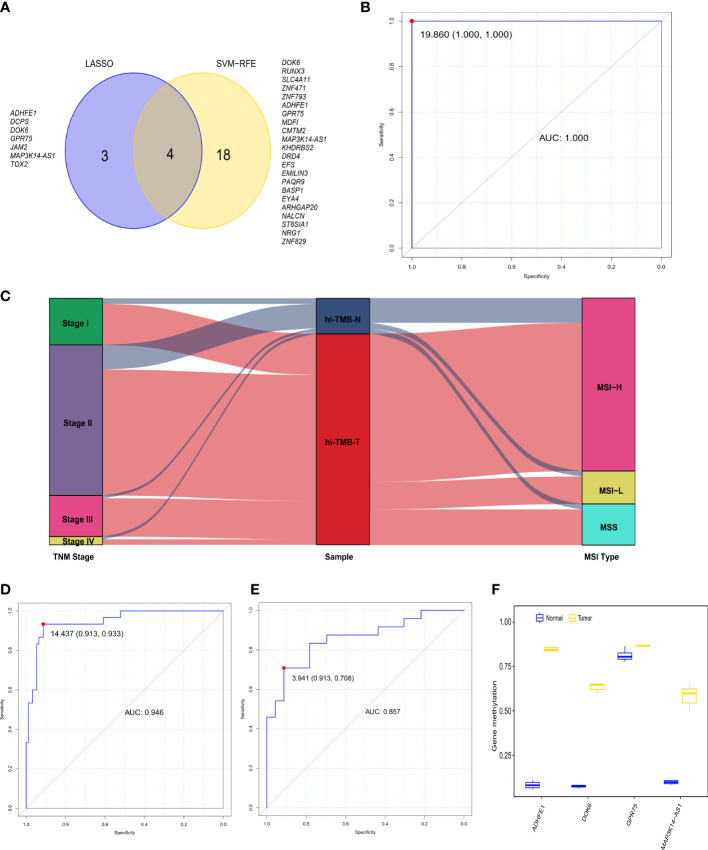
The diagnostic performance of the 4-gene methylation diagnostic score for colorectal tumor and adjacent normal tissues. **(A)** Screening of diagnostic markers *via* the Least absolute shrinkage and selection operator (LASSO) logistic regression algorithm and support vector machine-recursive feature elimination (SVM-RFE) algorithm of machine learning. **(B)** ROC curves of the diagnostic score constructed by the logistic regression model in the TCGA discovery set. **(C)** Alluvial diagram showing the changes of TNM stage, high TMB samples, and MSI type. **(D, E)** ROC curves of the diagnostic score validated in the GEO validation set A **(D)** and our in-house CRC validation set B **(E)**. **(F)** Methylation status of 4 genes in cell-free DNA samples from three colorectal cancer patients and four healthy controls in the GEO validation set C.

To further explore the differential methylation status of these 80 high-TMB CRC and 13 adjacent normal tissues in TCGA dataset and to identify methylation-related markers for high-TMB CRCs prediction, we screened 735 DMRs, covering 6012 methylation CpG sites, by comparing high-TMB CRCs with normal tissues. We further calculated the methylation levels of the DMRs at the promoter region, discovering 468 significant DMGs (**Supplementary Table S4**). According to the algorithms of machine learning, 7 DMGs were identified as key biomarkers by LASSO logistic regression algorithm (**Figure S2**), while 22 DMGs were determined as vital biomarkers by the SVM-RFE algorithm (**Figure S3**). Finally, *ADHFE1*, *DOK6*, *GRP75*, and *MAP3K14-AS1* were overlapping hypermethylated genes according to the two algorithms (**Figure 2A**). The AUCs of the four genes were 1.000, 1.000, 0.988, and 1.000, respectively, indicating that the four genes had a high accuracy in discriminating between high-TMB CRCs (N=80) and adjacent normal tissues (N=13) (**Figure S4**). Sensitivities and specificities under the different cut-off values of methylation levels were shown in **Supplementary Table S5**.

In addition, we constructed the DRS using a logistic regression method. The DRS was the sum of the methylation levels of the four genes at the promoter region weighted by the corresponding regression coefficient from the logistic regression model (**Supplementary Figure S5**): (130.82 × *ADHFE1*) + (52.36 × *DOK6*) - (74.95 × *GPR75*) + (96.49 × *MAP3K14-AS1*). The AUC of the DRS was 1.000 in TCGA CRC discovery set, with a sensitivity and specificity of 100% and 100% respectively (**Figure 2B**). Moreover, consistent with the previous reports that over 80% of MSI-high tumors display high TMB (39), we observed that 83.3% of high-TMB CRCs (N=90) exhibited MSI in the TCGA dataset (**Figure 2C**). We therefore used MSI to estimate high-TMB, screening122 possible high-TMB CRCs as a validation set in the GEO CRC datasets. We found the DRS showed robust performance (AUC = 0.946) in distinguishing 92 CRC tissues from 30 adjacent normal tissues in the GEO validation set A. The sensitivity and specificity of DRS were 93.3% and 91.3%, respectively (**Figure 2D** and **Figure S6**). Meanwhile, the DRS also demonstrated good performance (AUC = 0.857) in distinguishing 24 CRC tissues from 23 normal tissues in our CRC validation set. The sensitivity and specificity of DRS were 70.8% and 91.3%, respectively (**Figure 2E** and **Figure S7**). We further explored the methylation level of these four genes in cfDNA samples from the GSE122126 dataset. They were hypermethylated in all three CRC patients with metastases liver (3 out of 3) but not in healthy controls (0 of 4) (**Figure 2F**).

### Correlations between methylation of the four genes and immune infiltration cells

**Figure 3:**
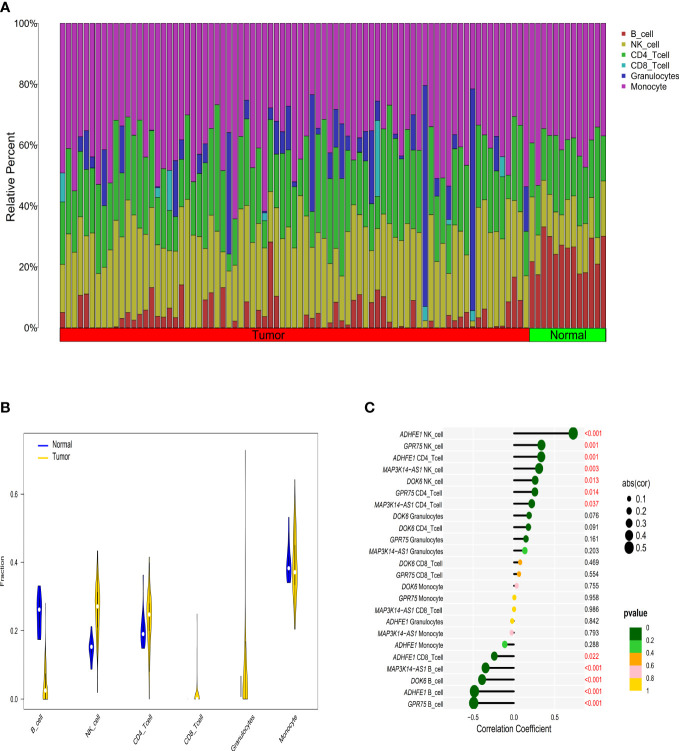
The relationship between immune cell infiltration characteristics and diagnostic biomarkers in CRC patients with high TMB. **(A)** The composition of tumor immune infiltrating cells in every high TMB sample. **(B)** Violin diagram of the difference in infiltration between the two groups of samples. **(C)** Correlation between diagnostic markers and immune infiltrating cells with the size of the dots representing the strength of the correlation between genes and immune cells and the color of the dots indicating the *P*-value.

According to the EpiDISH algorithm, we calculated the composition of infiltrating immune cells in high-TMB CRC and adjacent normal tissues (**Figure 3A**). Compared with normal tissues, CRC tumor tissues contained a higher proportion of NK cells (Wilcox test, *P*<0.001) and granulocytes (Wilcox test, *P*<0.001), but a relatively lower proportion of B cells (Wilcox test, *P*<0.001) (**Figure 3B**). In addition, we explored the correlations between methylation levels of the four genes and immune infiltrating cells, noting that *ADHFE1* showed significantly positive correlations with NK cell (r = 0.725, Spearman correlation test, *P*<0.001) and CD4+ T cell (r = 0.334, Spearman correlation test, *P*=0.001), but significantly negative correlations with CD8+ T cell (r = -0.239, Spearman correlation test, *P*=0.022) and B cell (r = -0.482, Spearman correlation test, *P*<0.001). *DOK6* displayed a positive correlation with NK cell (r = 0.260, Spearman correlation test, *P*=0.013) but a negative correlation with B cell (r = -0.389, Spearman correlation test, *P*<0.001). Meanwhile, *GPR75* displayed a strong positive correlation with NK cell (r = 0.336, Spearman correlation test, *P*=0.001) and CD4+ T cell (r = 0.256, Spearman correlation test, *P*=0.014), while *GPR75* displayed a strong negative correlation with B cell (r = -0.492, Spearman correlation test, *P*<0.001). *MAP3K14-AS1* displayed a positive correlation with NK cell (r = 0.308, Spearman correlation test, *P*=0.003) and CD4+ T cell (r = 0.218, Spearman correlation test, *P=*0.037), but a strong negative correlation with B cell (r = -0.346, Spearman correlation test, *P*<0.001) (**Figure 3C**).

### Construction and validation of methylation-based prognostic model in high-TMB CRCs

**Figure 4:**
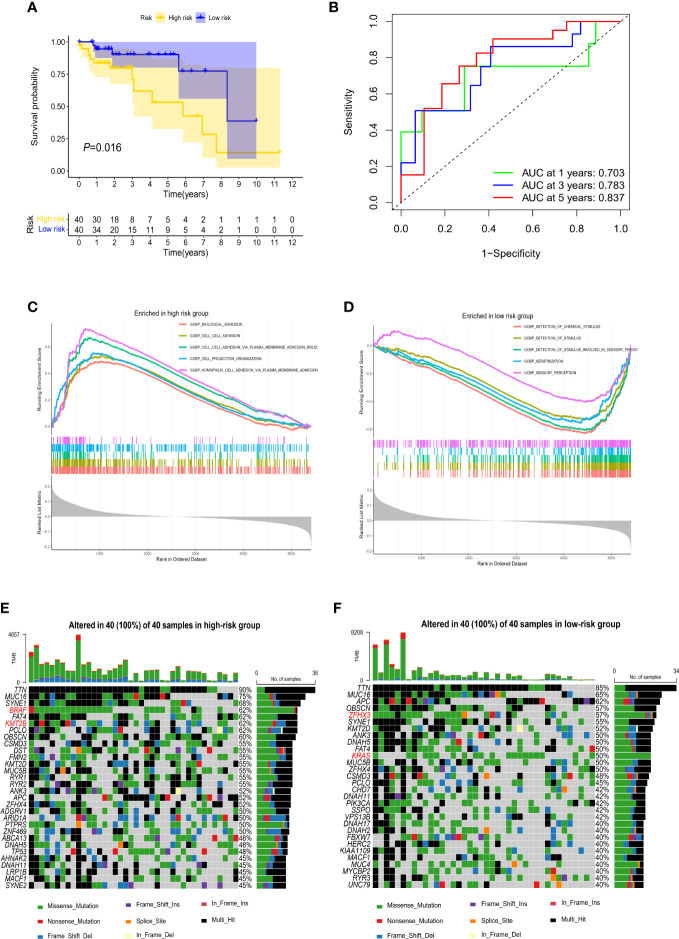
The prognostic performance of the 3-gene methylation prognostic score in CRC patients with high TMB. **(A)** The Kaplan–Meier survival curve on comparing survival between the high- and low-risk groups in the discovery set. **(B)** Time-dependent ROC curves analysis for the 1-, 3-, and 5-year survival prediction by prognostic model. **(C)** Gene sets enriched in the high-risk subgroup (*P* < 0.05, FDR < 0.25). **(D)** Gene sets enriched in the low-risk subgroup (*P* < 0.05, FDR < 0.25). The waterfall plot of tumor somatic mutation established by those in the high-risk group **(E)** and the low-risk group **(F)**. Mutated genes (rows top 30) are ordered by mutation rate. Each column represents individual patients. The right bar plot shows the proportion of each variant type.

We further estimated the association of CRCs’ OS with methylation levels of the above screened 468 DMGs between CRC tumor and adjacent normal tissues using univariate and multivariate Cox regression analysis. Three genes (*POU3F3*, *SYN2*, and *TMEM178A*) were strongly associated with the survival of 80 high-TMB CRCs (**Figure S8**). We then constructed a 3-gene methylation PRS model using TCGA CRC methylation data. The PRS = (2.221 × methylation level of *POU3F3*) - (0.635 × methylation level of *SYN2*) + (2.378 × methylation level of *TMEM178A*). In accordance with the PRS, we used the median as the cutoff value to divide CRCs into a high-risk group and a low-risk group. The Kaplan-Meier survival analysis showed high-risk group CRCs had poorer survival than low-risk group CRCs (log-rank test, *P*=0.016) (**Figure 4A**). In validation set, similar result was found in the COLONOMICS project dataset (log-rank test, *P*=0.001) (**Figure S9**). We further incorporated the detailed clinicopathologic characteristics of 80 high-TMB CRC patients in TCGA CRC cohort into Cox regression analyses. PRS were significantly associated with CRC prognosis in the univariate Cox regression analyses. Multivariate Cox regression analysis confirmed that 3-gene methylation PRS was an independent prognostic factor after adjusting for the TNM stage, T stage, and N stage. (HR= 2.499, 95%CI: 1.475- 4.232, Cox test, *P*=0.001) (**Table 2**). In addition, the predictive performance of the 3-gene methylation PRS showed that the AUCs were 0.703, 0.783, and 0.837 for 1-, 3-, and 5-year survival respectively by time-dependent ROC curve (**Figure 4B**).

**Table 2:** Univariate and multivariate Cox regression analysis of the 3-gene methylation signature.

Variables	Discovery set (N=80)				
	HR(95%CI)	*P*		HR(95%CI)	*P*
Univariate analysis			Multivariate analysis		
Age					
≥65 *vs* <65 years	1.368(0.482−3.879)	0.556	≥65 *vs* <65 years		
Sex					
Female *vs* Male	0.507(0.164−1.566)	0.237	Female *vs* Male		
TNM stage					
III+IV *vs* I + II	2.694(0.923−7.859)	0.070	III+IV *vs* I + II	4.601(0.494−42.860)	0.180
T Stage (Primary tumor)					
T3+T4 *vs* T1+T2	3.146(0.690−14.336)	0.139	T3+T4 *vs* T1+T2	3.047(0.619−15.003)	0.171
M Stage (Distant metastasis)					
M1 *vs* M0	2.073(0.464−9.237)	0.340	M1 *vs* M0		
N Stage (Regional lymph nodes)					
N1+N2 *vs* N0	3.054(0.910−10.244)	0.071	N1+N2 *vs* N0	0.352(0.026−4.710)	0.430
Microsatellite instability					
MSS+MSI-L *vs* MSI-H	0.829(0.287−2.391)	0.728	MSS+MSI-L *vs* MSI-H		
Tumor location					
Rectum *vs* Colon	1.248(0.279−5.574)	0.772	Rectum *vs* Colon		
3-gene methylation prognostic score					
High risk *vs* low risk	2.468(1.476−4.129)	0.001	High risk *vs* low risk	2.499(1.475−4.232)	0.001

To further clarify the biological function of high-risk and low-risk CRCs, we conducted GSEA analysis, observing that the gene sets of the high-risk CRCs were enriched in cell adhesion (**Figure 4C**) whereas the gene sets of the low-risk CRCs were enriched in stimulus (**Figure 4D**). The detailed results of GSEA are summarized in **Supplementary Table S6**. We further identified that the mutation rates of *TTN* and *MUC16* were higher than 60% in both low- and high-risk CRCs in screening genome-wide mutation. *BRAF*, *KMT2B* gene mutations were more common biomarkers in the high-risk CRCs (**Figure 4E**), whereas *ZFHX3* and *KRAS* gene mutations were more common biomarkers in the low-risk CRCs (**Figure 4F**).

### Immune characteristics and the benefit of ICI therapy in high-TMB CRCs

**Figure 5:**
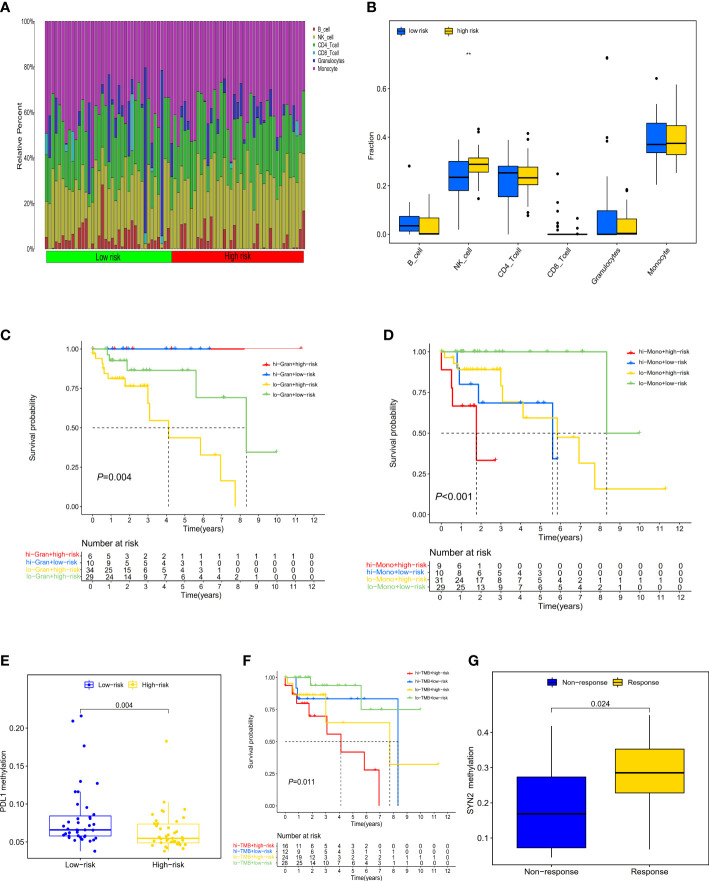
Distribution and characteristics of immune cells infiltration and the prognostic value of risk score in patients with ICI therapy. **(A)** The composition of tumor immune cells infiltration in every high- and low-risk sample. **(B)** Differences in six type of infiltration cells between the two groups of samples (**P < 0.01. **(C)** The survival of patients with high (low) risk and high (low) infiltrating granulocytes levels. **(D)** The survival of patients with high (low) risk and high (low) infiltrating monocyte levels. **(E)** Differences in *PD-L1* methylation between high- and low-risk score subgroups. **(F)** The survival of patients with high (low) risk and high (low) TMB. **(G)** Differences in *SYN2* methylation between distinct immunotherapy clinical response groups.

Evaluating the landscape of the tumor microenvironment (TME) could contribute to improving the effectiveness of immunotherapies for CRC. We therefore further evaluated the composition of infiltrating immune cells in high- and low-risk CRCs by the EpiDISH algorithm, revealing that high-risk CRCs contained higher levels of infiltrating NK cells than low-risk CRCs (Wilcox test, *P*<0.01) (**Figures 5A, B**). We estimated the associations of high-TMB CRC prognosis with the infiltrating levels of six immune cells. We observed that low infiltrating granulocyte (log-rank test, *P*=0.017) or monocyte (log-rank test, *P*=0.002) levels showed a poorer survival rate than high infiltrating granulocytes or monocyte levels (**Figures S10**, **S11**). Additionally, we observed a significant survival advantage in high-TMB CRCs with high infiltrating granulocyte levels regardless of risk score by conjoint survival analysis with PRS (log-rank test, *P*=0.004) (**Figure 5C**). Meanwhile, high-TMB CRC patients with a low-risk score and low infiltrating monocyte levels also displayed a better survival advantage (log-rank test, *P*<0.001) (**Figure 5D**).

To explore the prognostic performance of PRS in clinical response to anti-*PD-1*/*PD-L1* immunotherapy, we first compared the difference of *PD-L1* methylation levels between high- and low-risk CRCs, observing higher *PD-L1* methylation levels in low-risk CRCs (**Figure 5E**). The Kaplan-Meier survival analysis showed that low-risk CRCs with low *PD-L1* methylation had a great survival advantage compared with other groups (log-rank test, *P*=0.001) (**Figure S12**). Furthermore, we did not identify a significant difference in TMB between low- and high-risk CRCs (Wilcox test, *P*=0.07) (**Figure S13**). In the high-TMB CRC patients, we used the best cutoff value (35.71 mutations/Mb) to divide CRCs into high- and low-TMB groups. In the survival analysis, we observed that regardless of high- and low-TMB, low-risk PRS showed both a favorable survival (log-rank test, *P*=0.011) (**Figure 5F**).

In order to evaluate the predictive performance of PRS in other tumors, we investigated whether the three-gene methylation PRS could predict a clinical response to ICI therapy in the metastatic melanoma immunotherapy cohort (GSE175699). 44 metastatic melanoma patients in both the high- and low-risk groups did not exhibit significant clinical benefits from ICI therapy (Wilcox test, *P*=0.69) (**Figure S14**); however, we separately explored the effect of the methylation level of these three genes (*POU3F3*, *SYN2*, and *TMEM178A*) on immunotherapy, finding that patients with low *SYN2* methylation levels showed a poorer clinical response to ICI therapy than those with high methylation levels (Wilcox test, *P*=0.024) (**Figure 5G**).

## Discussion

Previous studies have demonstrated that CRCs with dMMR/MSI-H exhibited dense immune infiltrate and tended to respond to ICI therapy, especially in CRCs with metastatic dMMR/MSI-H. CRCs with MSI-H only accounted for 15% of CRCs, however. In recent years, TMB has been recognized as a potential biomarker for stratifying CRCs’ response to ICI therapy. Moreover, as well as 83% of MSI CRC patients showing high TMB, some MSS CRC patients also showed a high TMB while possibly heavily infiltrated by immune cells and responsive to ICI therapy (40). High TMB may therefore be more widely used than dMMR/MSI. In addition, the TMB landscape from 100,000 cancer genomes found that only 16% of tumor patients with high TMB were classified as MSI-H (39), indicating that high TMB may be involved in different tumor stages rather than just metastatic stages; hence, TMB may be an effective biomarker for a clinical response to immunotherapy in CRC patients. The high cost of WES sequencing and the inconsistent cut-off values of high TMB, however, limit the clinical application of TMB. TMB combined with other biomarkers may therefore be an efficient mean of screening CRCs with a clinical response to immunotherapy.

Interestingly, recent studies proposed that DNA methylation-based pattern change was associated with *PD-L1* expression and TMB, which may serve as a predictive biomarker to select lung cancer patients with a clinical response to ICI therapy (23, 26). In this study, we identified a significant positive correlation between TMB and DNA methylation in CRCs, especially CRC-related global methylation changes in high-TMB CRC tumor tissues. Although earlier studies reported a positive correlation between DNA hypomethylation and cell karyotype instability and higher mutation rates, these hypomethylated regions did not perform a biological function (41). Biological functions of CRC-related DNA methylations were mainly hypermethylation of CpG islands in the promoter region accompanied by multiple gene mutations, including *BRAF* mutation (20, 42). Moreover, due to the robustness and reasonable cost of the DNA methylation method, we also estimated the potential predictive effect of DMG together with TMB in screening CRCs with a clinical response to ICI therapy by systematically integrating DNA methylation profiling and gene mutation data.

To further explore the correlation between DNA methylation and high TMB, we identified 468 DMGs genes that were significant differential methylated regions in this study. According to the algorithms of machine learning (LASSO regression and SVM-RFE), *ADHFE1*, *DOK6*, *GRP75*, and *MAP3K14-AS1* were selected to construct a diagnostic model and were successfully validated in GEO and our CRC samples datasets. Additionally, CRCs with high TMB showed high infiltrating NK cells and low infiltrating B cell levels. These four genes were also positively correlated with NK cells. It may therefore be necessary to further explore the biological functions of these four genes in CRC-related NK cells. Additionally, *ADHFE1* methylation at the promoter region has been reported as a potential early diagnostic biomarker with high sensitivity and high specificity in CRC tumor tissues, stool, and adenomas tissues (43–45). A recent study found that hypermethylation of *ADHFE1* promotes cell proliferation by modulating cell cycle progression in CRC (46). These findings may explain the high accuracy of *ADHFE1* hypermethylation in discriminating high-TMB CRCs and normal controls. A previous study reported that *GPR75* hypermethylation may serve as a diagnostic biomarker for CRC in African-Americans, and that it was very closely related to the insulin/TGF-β1 pathway (47). Moreover, we found in a recent study (48), surprisingly, that hypermethylation of the *MAP3K14-AS1* gene in cfDNA samples can be used to monitor treatment response in CRCs with metastasis. All of the above results encouraged us to find more CRC patients with high TMB who were responsive to ICI treatment. *DOK6* (Docking Protein 6), a member of the *DOK* family of intracellular adaptors, was associated with Hirschsprung Disease 1; however, *DOK6* has not been reported as a diagnostic marker for CRC, and the biological mechanism in CRC has not yet been discovered.

We further identified three genes (*POU3F3*, *SYN2*, and *TMEM178A*) as biomarkers for prognostic evaluation in high TMB CRC patients, and we built and verified a prognostic risk model consisting of these three genes. The 3-gene methylation PRS was an independent predictor for high-TMB CRC prognosis. In addition, *BRAF* was frequently mutated in the high-risk PRS CRCs, whereas *KRAS* was frequently mutated in the low-risk PRS CRCs. This result supports the previous finding that DNA hypermethylation was accompanied by high *BRAF* mutation. Moreover, high-TMB CRC patients with low-risk scores exhibited hypermethylation of *PD-L1*, indicating a potential response to anti-*PD-1*/*PD-L1* therapy. High-TMB CRCs with low-risk PRS had a significant survival advantage. The 3-gene methylation PRS may be a potential biomarker for high-TMB CRC prognosis and clinical response assessment of immunotherapy. Further, we observed a great survival advantage in high-TMB CRC patients with high infiltrating granulocytes regardless of the high- or low-risk score, and in high-TMB CRC patients with low infiltrating monocyte and low-risk PRS. The 3-gene methylation PRS combined with immune infiltrating cells may be a surrogate marker for the prognosis of high-TMB CRCs.

To the best of our knowledge, this is the first study to investigate a direct correlation between DNA methylation and TMB in CRCs. By integrating analysis of genomic and epigenetic data, we identified and constructed methylation-based early diagnostic and prognostic models for high-TMB CRC, estimating their predictive effect for immunotherapy in high-TMB CRCs. Moreover, the diagnostic model has been successfully validated in our CRC dataset, while these methylation markers have also been reported in other CRC tumor tissues, cfDNA, and fecal samples. The combination of PRS and infiltrating cells could effectively identify CRC patients with a significant survival advantage and improve the precision management of CRC ICI therapies, while the three-gene methylation PRS may be a surrogate marker for immunotherapy. We nevertheless acknowledge possible limitations in the present study. Firstly, although hypermethylation of these diagnostic biomarkers was observed in CRC cfDNA samples, whether the diagnostic panel can be applied as a non-invasive biomarker should be further determined in larger studies. Secondly, as the thresholds used for TMB in different tumor types have obvious differences, further studies are warranted to define high TMB and low TMB using cancer-specific thresholds or uniform thresholds. Thirdly, in the PRS validation set, there are currently only 38 high-TMB CRCs, and only two cases in the low-risk group. A large number of prospective high-TMB CRCs may be needed to validate our prognostic methylation model for the future. Finally, the current study limits accurate assessment of the association between prognostic methylated PRS and immunotherapy response in high TMB CRC patients due to the lack of CRCs with immunotherapy. Subsequent studies may need to collect immunotherapy CRC patients to fill in this gap in the future.

In summary, this study discovered and validated that *ADHFE1*, *DOK6*, *GRP75*, and *MAP3K14-AS1* gene methylation hold great promise as a diagnostic panel for screening CRCs with high TMB. *POU3F3*, *SYN2*, and *TMEM178A* methylation could be a promising marker for improving the clinical prognostic evaluation of CRCs with high TMB. In addition, PRS combined with high granulocytes or low monocyte infiltrating levels represented favorable survival for high-TMB CRCs. More comprehensive studies with larger sample sizes and *PD-L1* clinical outcomes are warranted to shed light on the effect of DNA methylation patterns on the diagnosis and prognosis of high-TMB CRCs and the clinical response of immunotherapy.

## Data availability statement

The data presented in the study are deposited in the Genome Sequence Archive (GSA) repository, accession number HRA003126.

## Ethics statement

The study was ethically approved by the Institutional Ethics Committee of Harbin Medical University. The patients/participants provided their written informed consent to participate in this study.

## Author contributions

DH and FH designed and supervised the research project and revised the manuscript until submitting it. HHuang and WC performed research and drafted the manuscript. ZL, LK, and YF performed the experiment and analyzed data. XL, LYang, and XZ collected the data and analyzed the data. XS, LYuan, and TL revised the manuscript. YW, YZ, YC, and PS re-analysis results and interpretation. GL, PP, JZ, and HHu performed the figures and edited the data. XS, LY, and TL revised the manuscript. All authors read and approved the final manuscript. All authors contributed to the article and approved the submitted version.
