# Supplementary material for: DNA methylation-based patterns for early diagnostic prediction and prognostic evaluation in colorectal cancer patients with high tumor mutation burden
Source: Front Oncol. 2023 Jan 13;12:1030335. doi: 10.3389/fonc.2022.1030335 (PMC9880489; doi:10.3389/fonc.2022.1030335)
Supplement: Supplementary file 1 [file DataSheet_1.pdf]

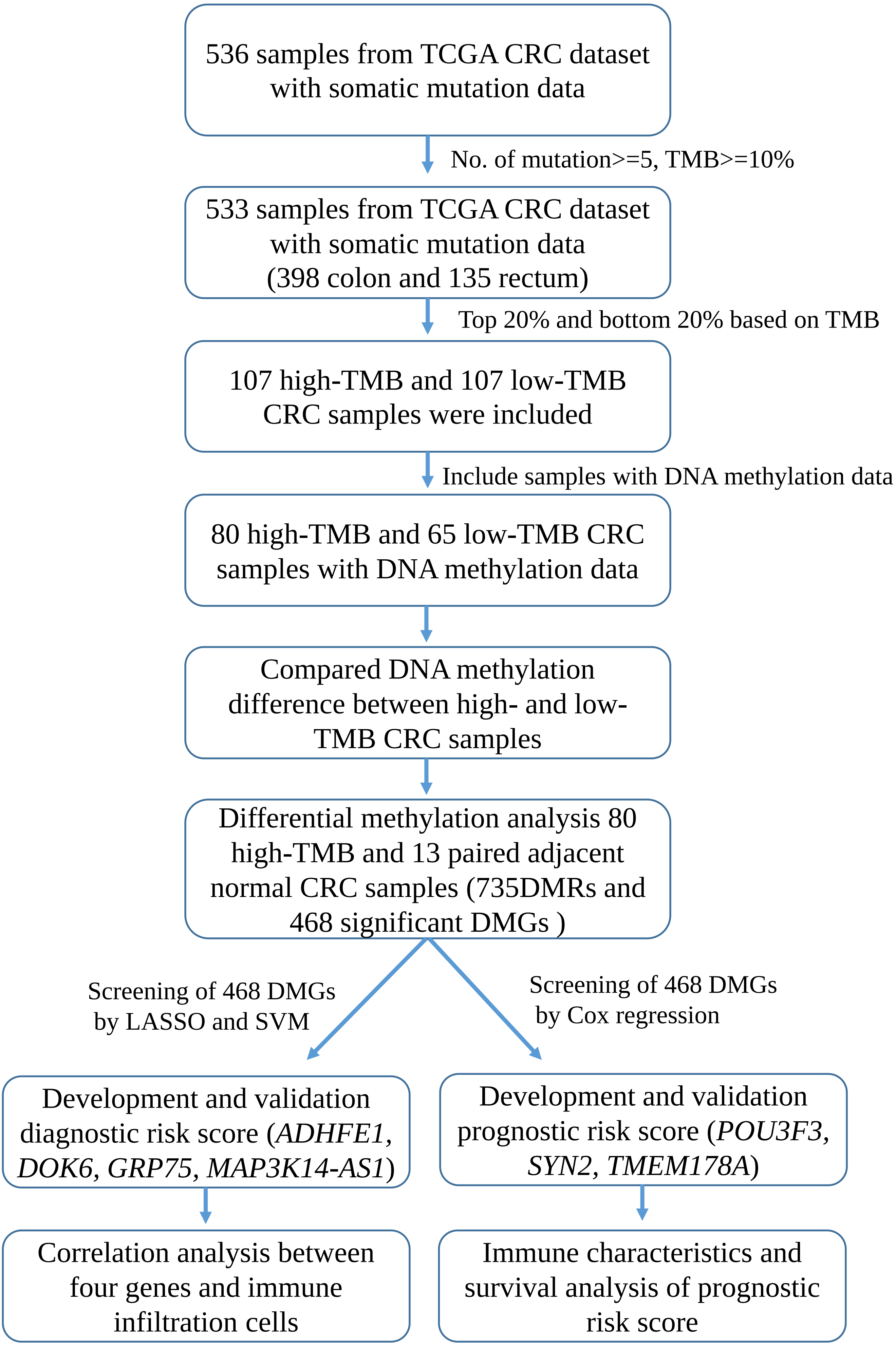

Figure S1. Flow chart of research. CRC, Colorectal cancer; TMB, Tumor mutation burden; DMRs, Differential methylation regions; DMGs, Differential methylated genes; LASSO, Least absolute shrinkage and selection operator; SVM, support vector machine.

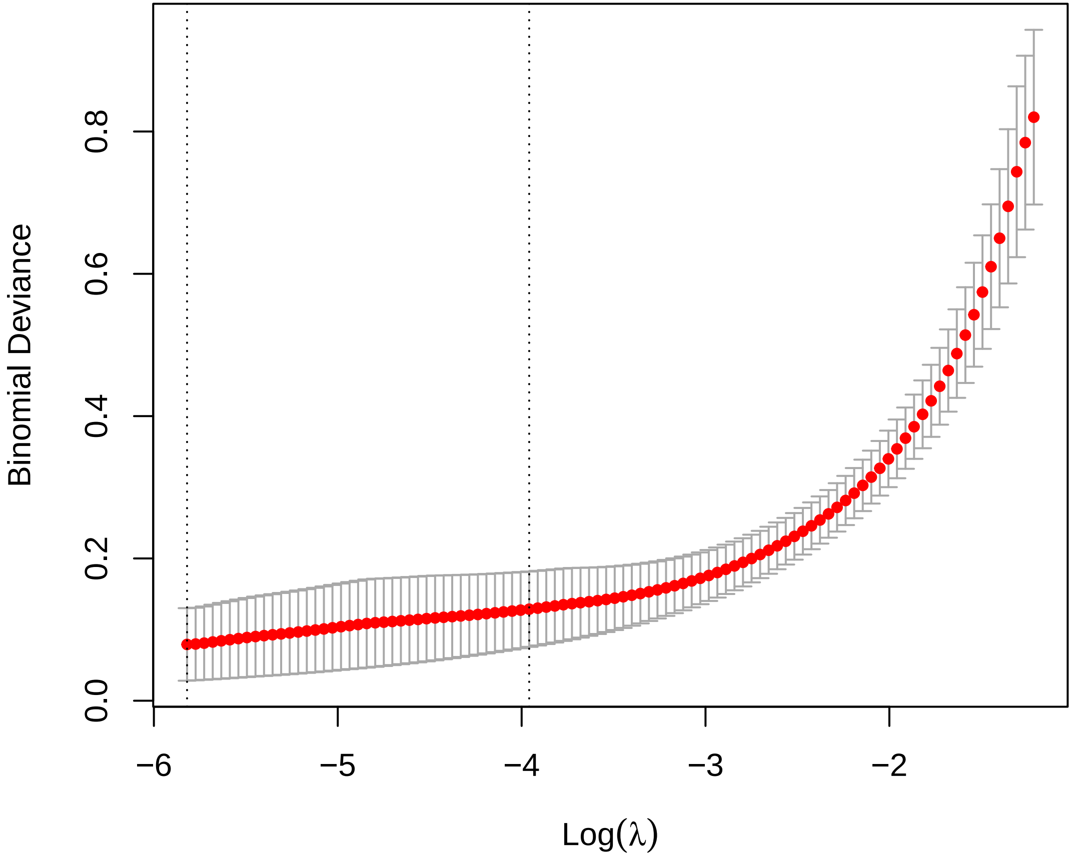

Figure S2. Least absolute shrinkage and selection operator (LASSO) logistic regression algorithm to screen diagnostic markers. Tuning parameter (λ) selection in the LASSO model used cross-validation via the maximum criteria. The dotted vertical lines were drawn at the optimal values using the maximum criteria and the one standard error of the maximum criteria.

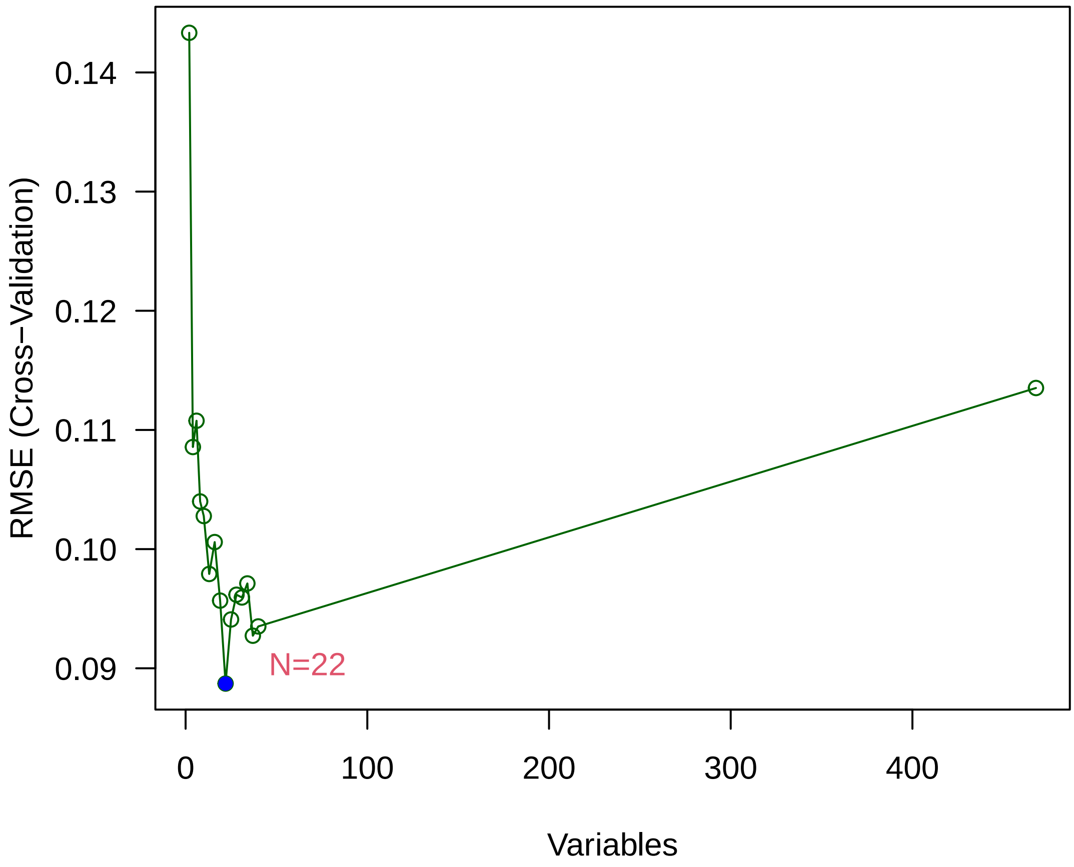

Figure S3. Support vector machine recursive feature elimination (SVM-RFE) algorithm to screen diagnostic biomarkers.

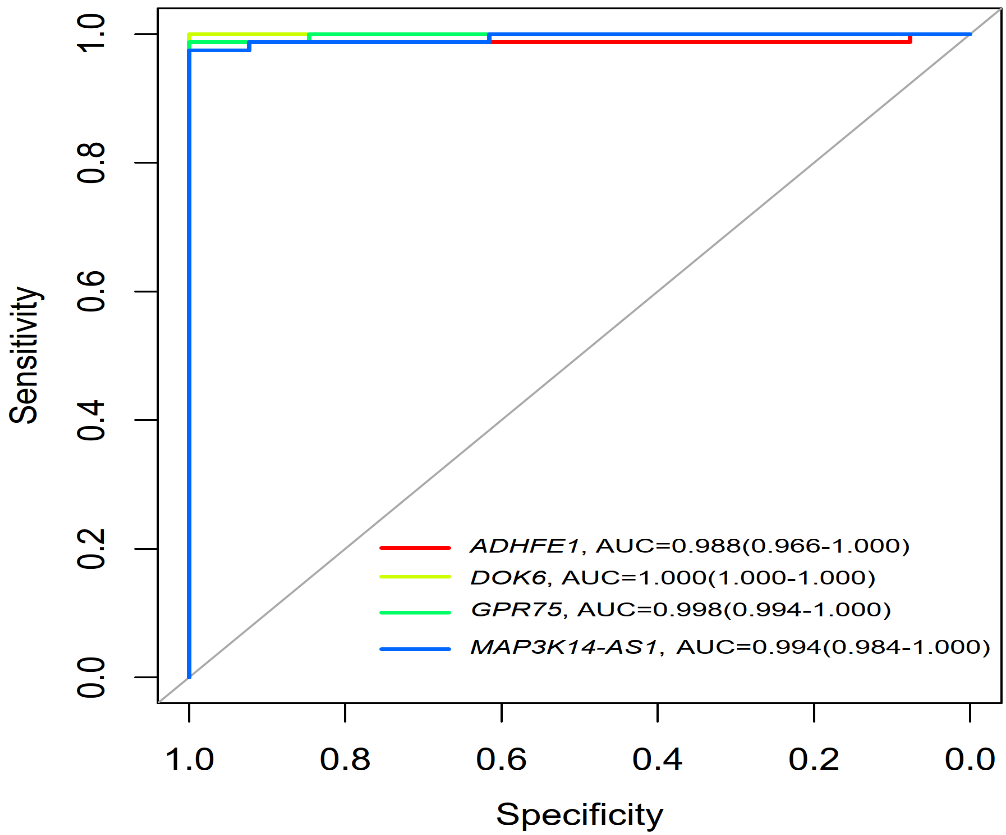

Figure S4. The ROC curve of the diagnostic markers of four genes in TCGA discovery set.

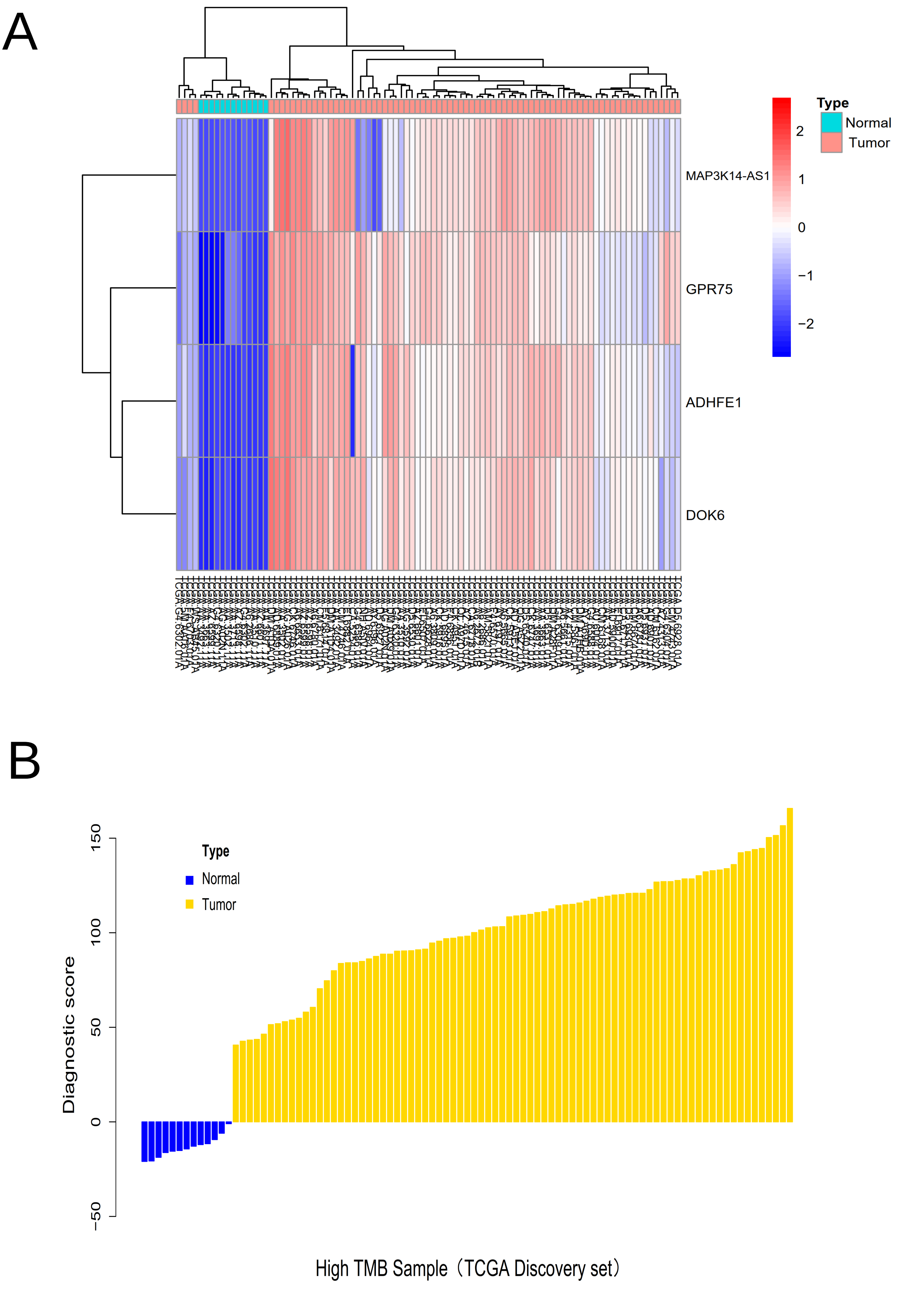

Figure S5. The 4-gene diagnostic score between CRC and normal tissues in the TCGA discovery set. (A) Unsupervised hierarchical clustering and heatmap of four genes and (B) the waterfall plot illustrated the 4-CpG diagnostic score.

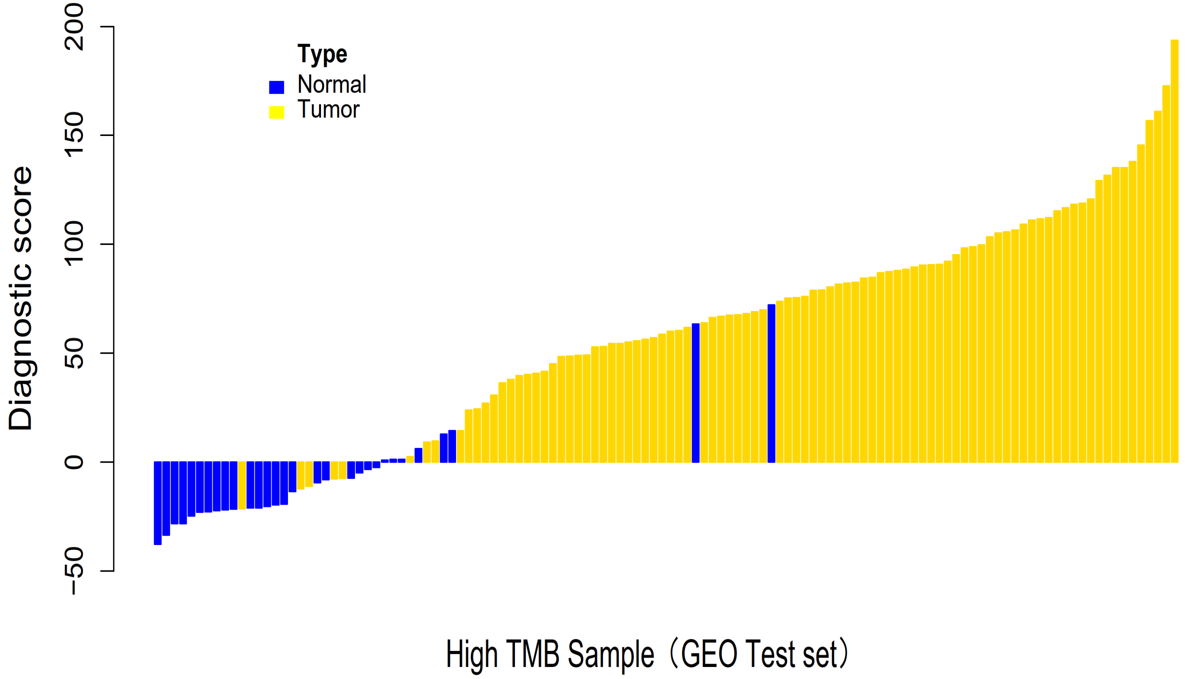

Figure S6. The 4-gene diagnostic score between CRC and normal tissues in the GEO test set. The waterfall plot illustrated the 4-CpG diagnostic score.

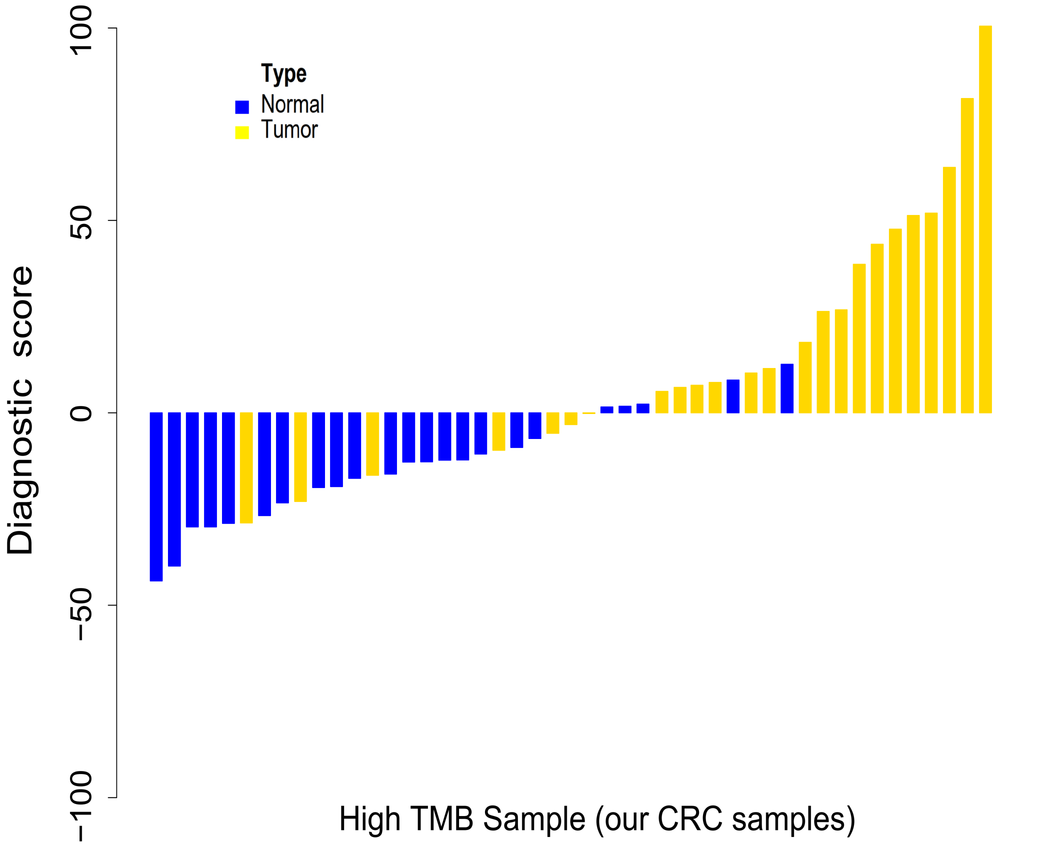

Figure S7. The 4-gene diagnostic score between CRC and normal tissues in our CRC samples. The waterfall plot illustrated the 4-CpG diagnostic score.

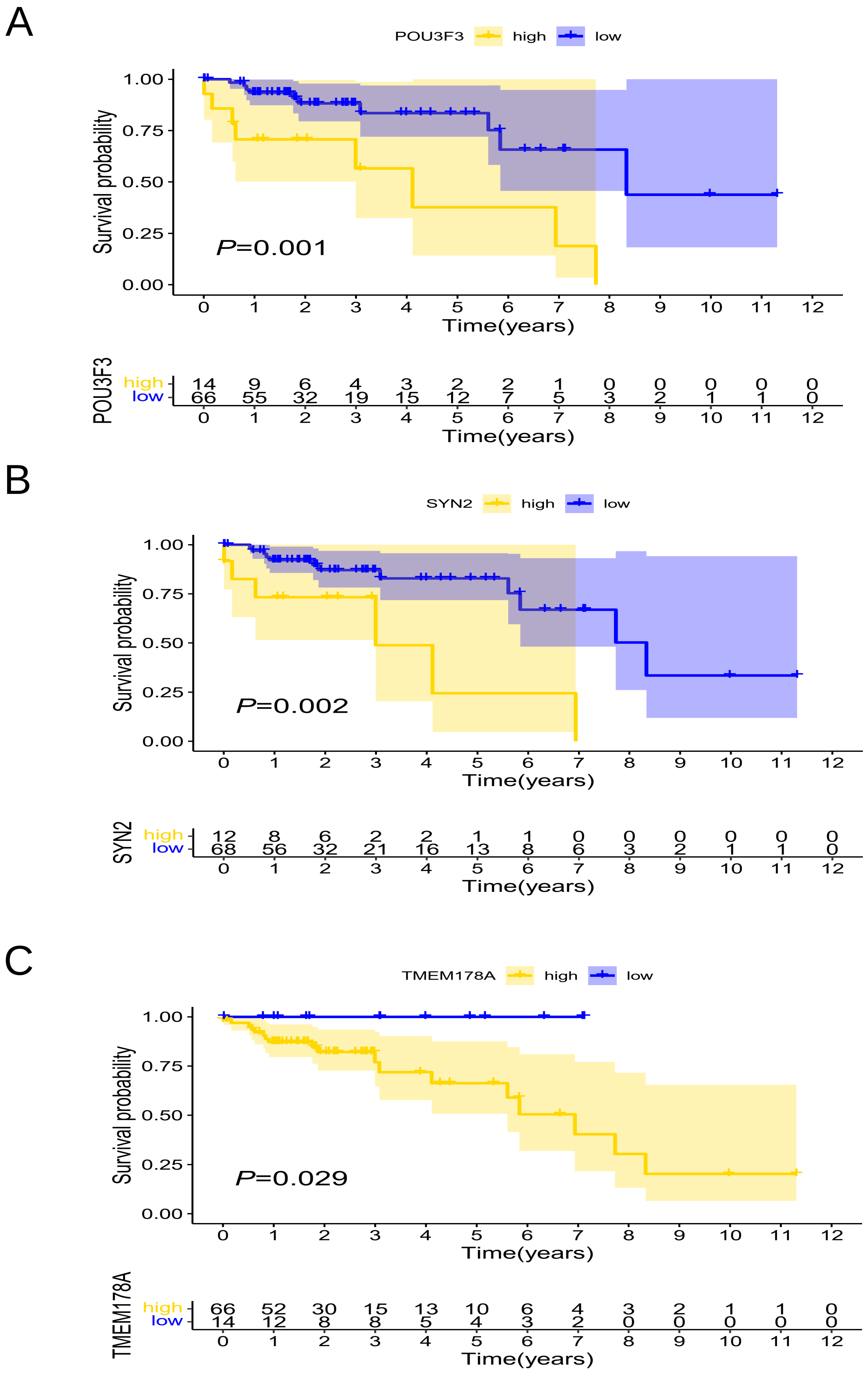

Figure S8. The Kaplan–Meier survival curve on comparing survival between the high methylation and low methylation levels of *POU3F3* (A), *SYN2* (B), and *TMEM178A* (C).

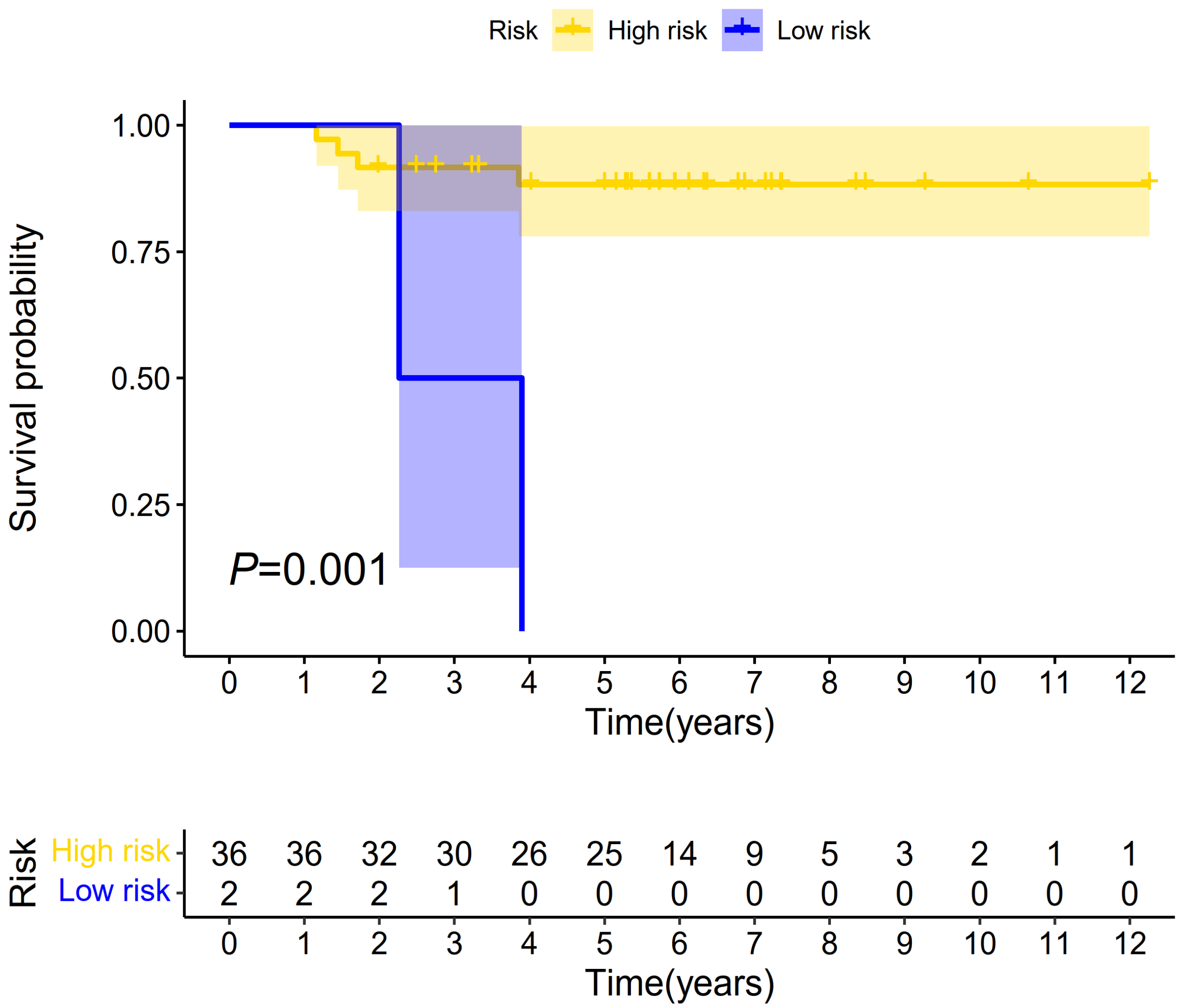

Figure S9. The Kaplan–Meier survival curve on comparing survival between the high- and low-risk groups in the validation set.

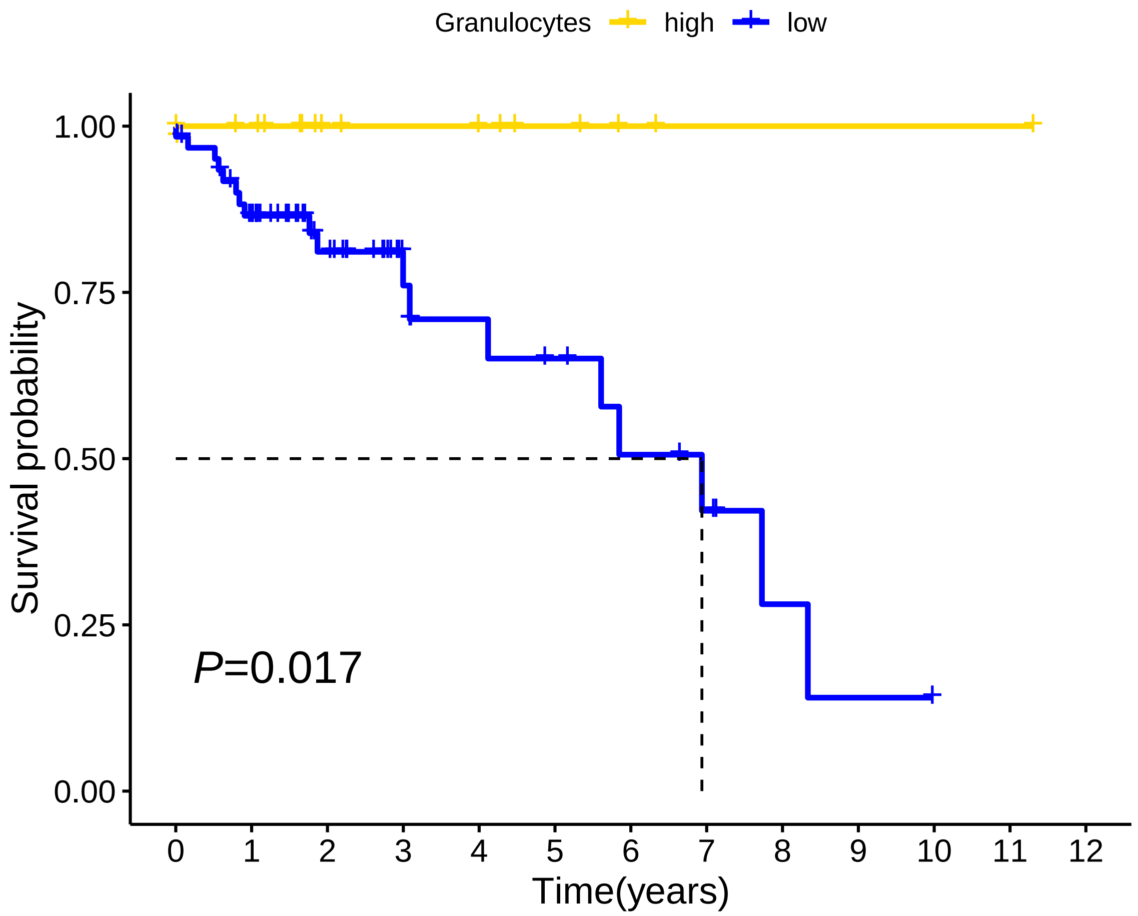

Figure S10. The Kaplan–Meier survival curve on comparing survival between the high infiltrating granulocytes and low infiltrating granulocytes levels.

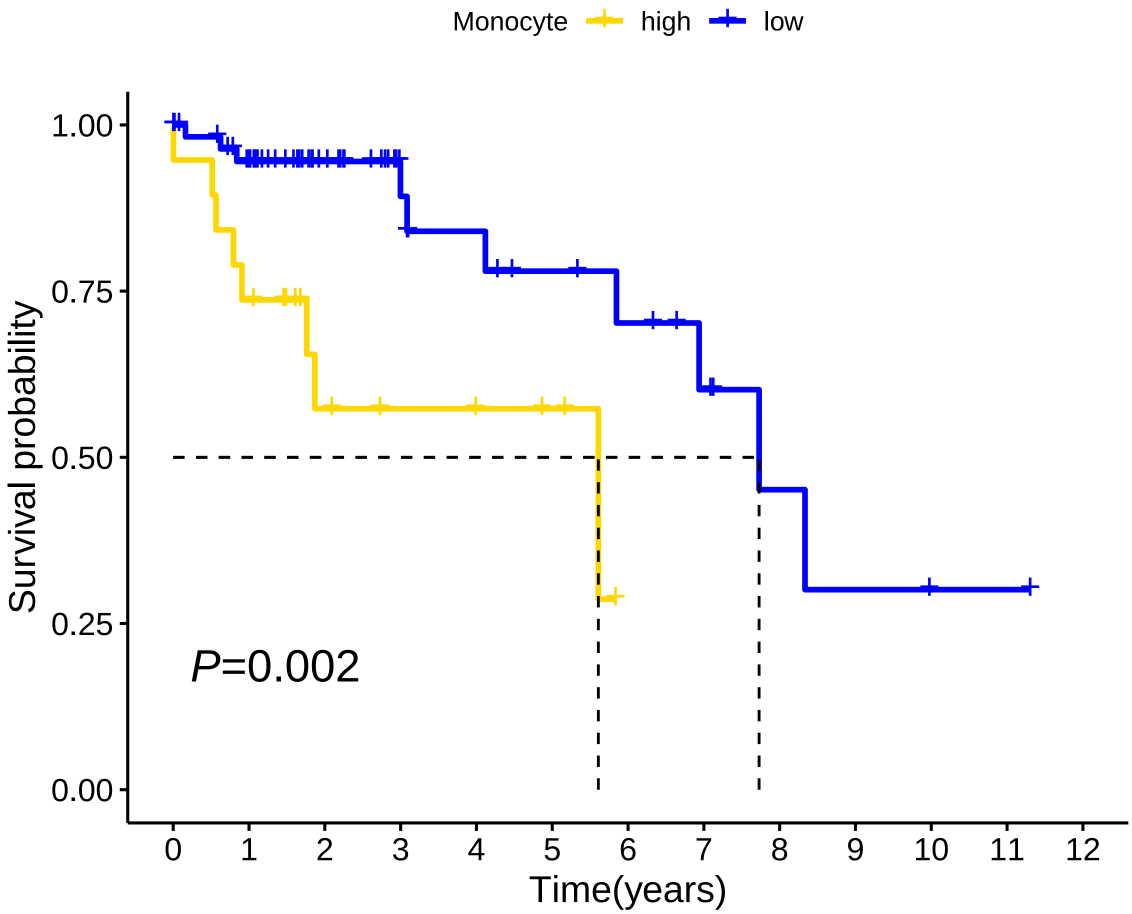

Figure S11. The Kaplan–Meier survival curve on comparing survival between the high infiltrating monocyte and low infiltrating monocyte levels.

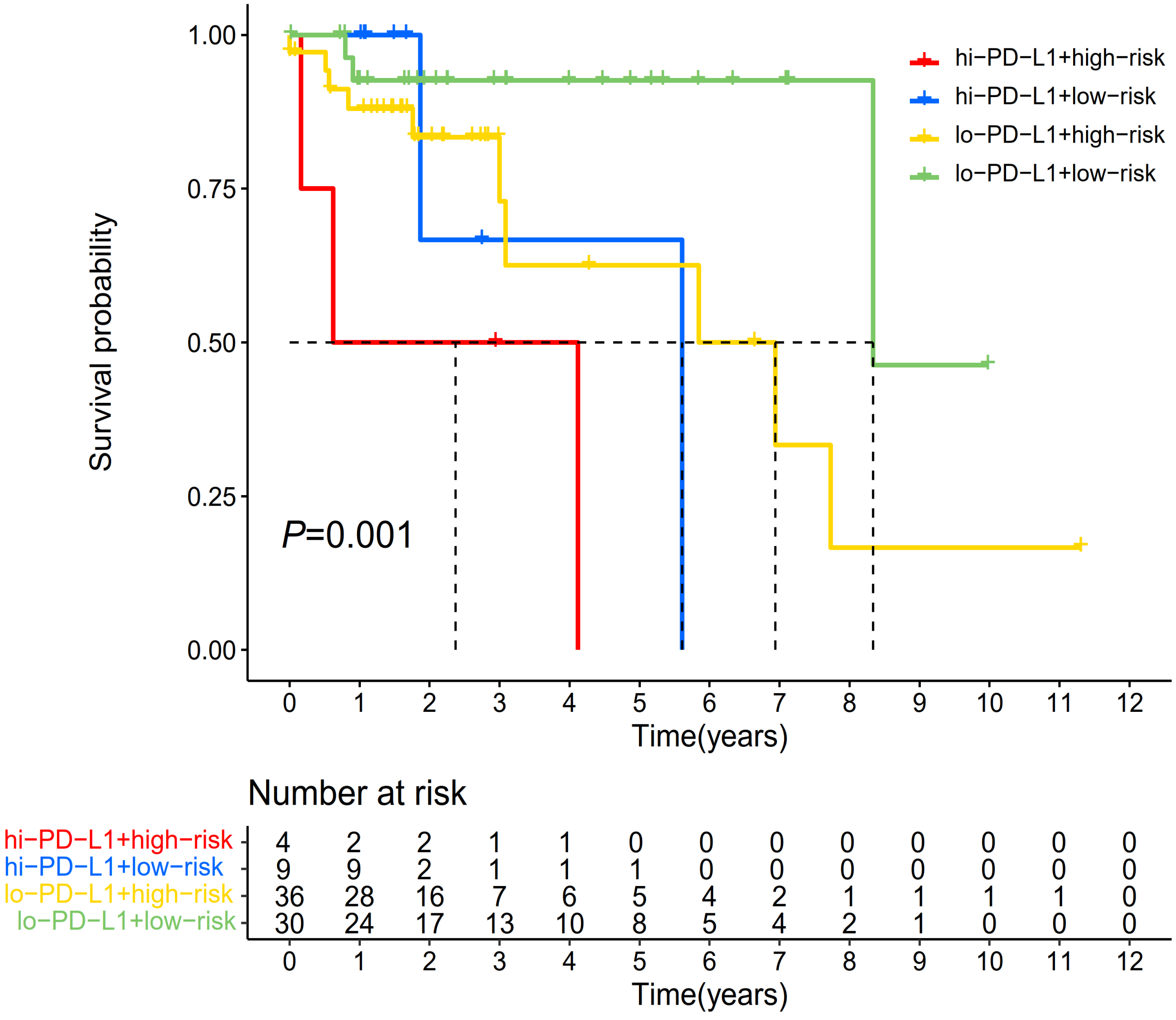

Figure S12. The survival of patients with high (low) prognostic score and high (low) PD-L1

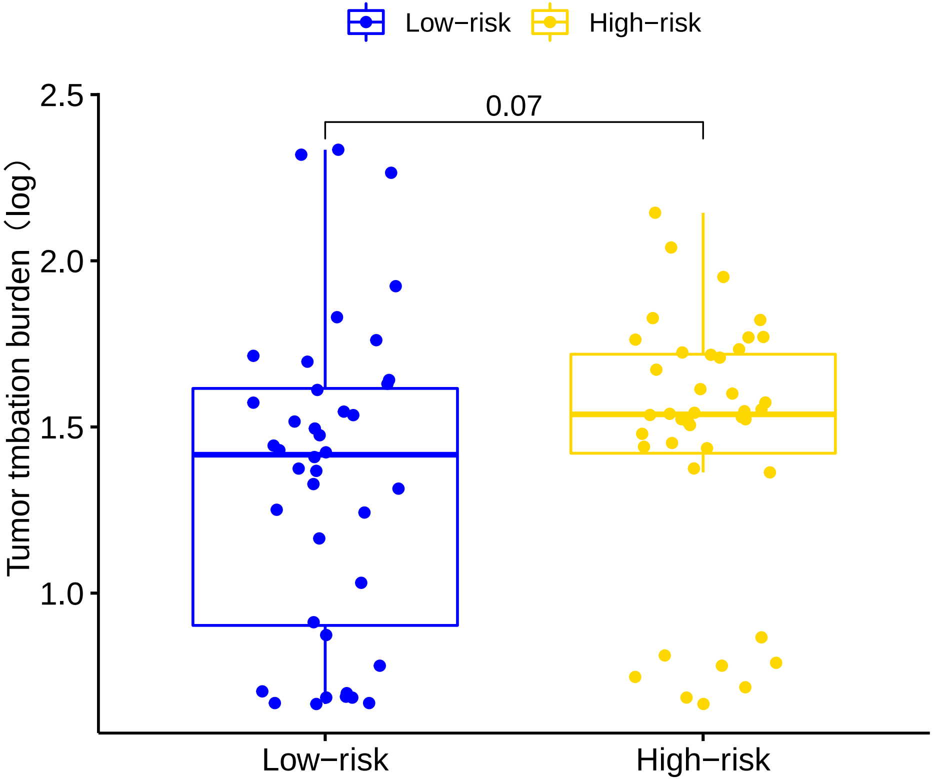

Figure S13. Differences in TMB between high- and low-risk score subgroups

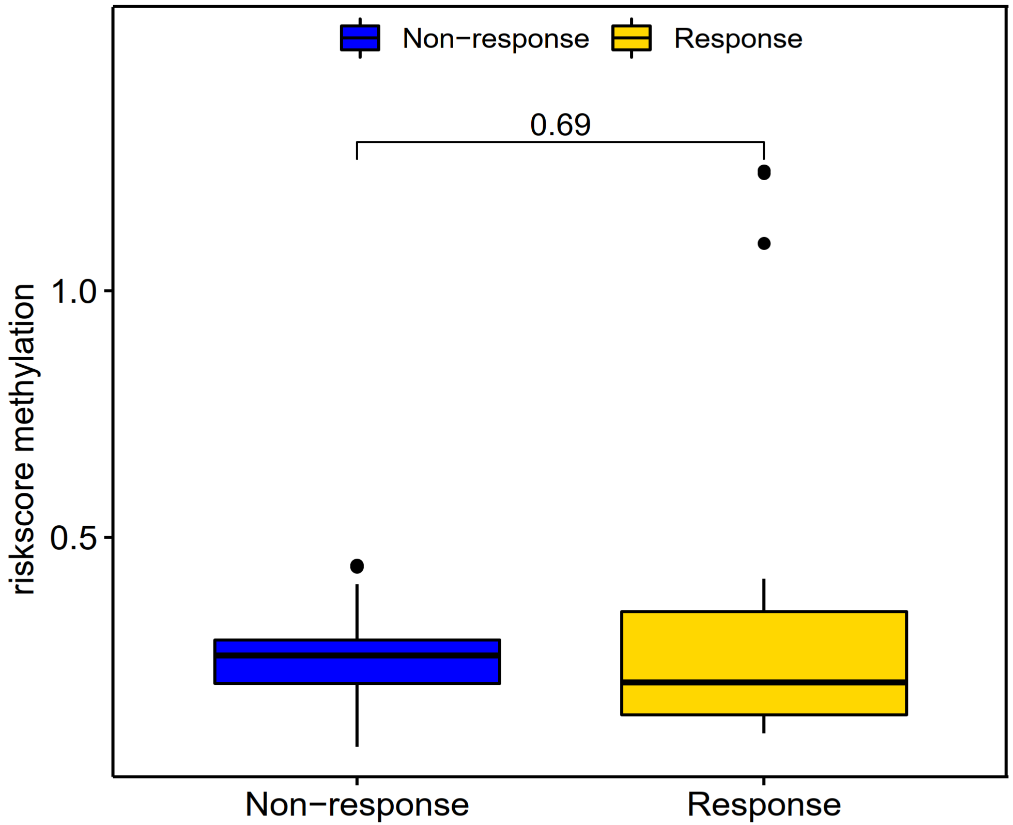

Figure S14. Differences in PRS methylation between distinct immunotherapy clinical response groups.
